# Supplementary figures and images for: Host response to bacteria induces a shift towards the enteroendocrine cell lineage in the murine enteroid model
Source: PLoS Pathog. 2026 Mar 23;22(3):e1014069. doi: 10.1371/journal.ppat.1014069 (PMC13052877; doi:10.1371/journal.ppat.1014069)

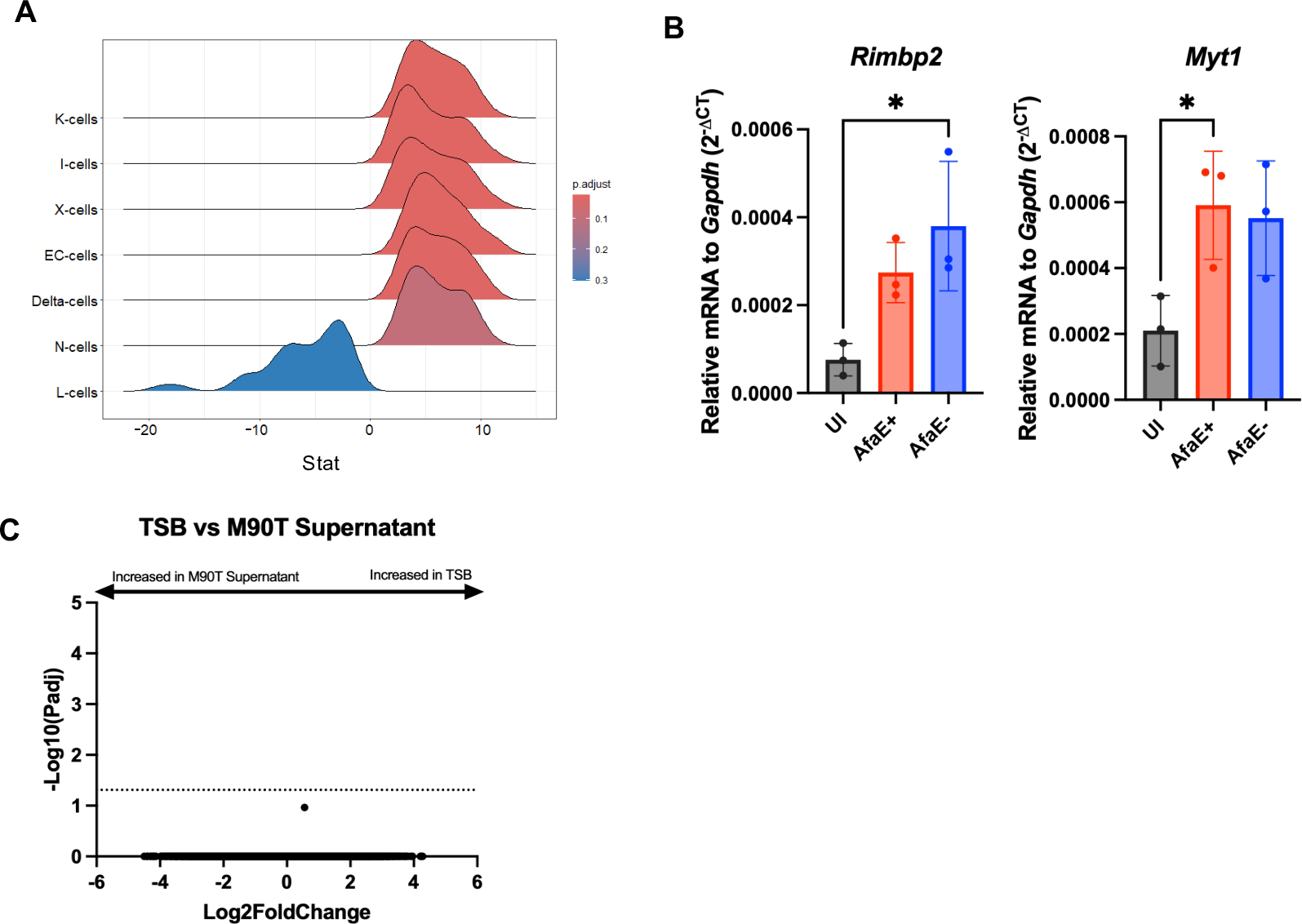

Supplement: S4 Fig — (A) GSEA was performed using transcriptomic data from Nlrc4-/- enteroids infected with S. flexneri M90T at an MOI of 50 compared to UI controls. Subtype-specific EEC gene signatures were defined according to Gehart et al. [39]. (B) qPCR analysis of Nlrc4-/- enteroids infected with DH5α E. coli transformed with an afimbrial adhesin protein AfaE at an MOI of 50 for 4 hours. (C) Volcano plot of differentially expressed genes between TSB control and M90T supernatant-treated Nlrc4-/- Intestinal organoids. Statistical analysis in panel B was performed using one-way ANOVA. Data represent mean ± SD, with p < 0.05 considered statistically significant. Data are representative of at least three independent biological replicates. (TIF) [file ppat.1014069.s004.tif]
