# Supplementary material for: Host response to bacteria induces a shift towards the enteroendocrine cell lineage in the murine enteroid model
Source: PLoS Pathog. 2026 Mar 23;22(3):e1014069. doi: 10.1371/journal.ppat.1014069 (PMC13052877; doi:10.1371/journal.ppat.1014069)
Supplement: Attachment — Submitted filename: Response to reviewers.docx [file ppat.1014069.s007.docx]

**Rebuttal letter PLOS Pathogens**

Dear editor and reviewers,

This document contains a point-by-point response to the comments provided on our manuscript titled “Host response to bacteria induces a shift towards the enteroendocrine cell lineage in the murine enteroid model”. We have also attached the revised manuscript with changes tracked so you may see how each new experiment is embedded into the text. We thank reviewers for their attentiveness and suggestions to the manuscript, all of which have strengthened the new submission greatly. Below we have summarized all the new experiments done for the revised manuscript as per the reviewer’s suggestions, all of which will be discussed in the point-by-point response:

- Immunofluorescence and quantification of enteroendocrine cells in *Nlrc4*^-/-^ enteroids infected with *S. flexneri.* The results show that there is an increase in the number of enteroendocrine cells upon infection with M90T and BS176 (Fig. 6 G-H).
- CFU kinetics of M90T and BS176 in HeLa cells and *Nlrc4*^-/-^ enteroids. These experiments revealed there is replication of M90T in the classical infection model of HeLa cells, but there is limited or no bacterial replication in *Nlrc4*^-/-^ enteroids (Fig. 2A-B).
- Immunofluorescence and Z-stacks of *Nlrc4*^-/-^ enteroids incubated with beads. These experiments revealed that there was uptake of the fluorescent beads into the organoids (Fig. 2F).
- Immunofluorescence and Z-stacks of *Nlrc4*^-/-^ enteroids infected with *E. coli*. This revealed that there was uptake of *E. coli* into the organoids (Fig. 2E).
- CFUs of *Nlrc4*^-/-^ enteroids infected with *E. coli.* This revealed that there were *E. coli* that entered the cells and were protected from the gentamicin(Fig. 2D).
- qPCR of *Nlrc4*^-/-^ enteroids infected with *E. coli*. The results showed that there was an increase of enteroendocrine cell markers even with *E. coli* infection (Sup. Fig 4B).
- Repeating inflammasome western blots of *Nlrc4*^-/-^ enteroids infected with M90T and BS176 in the presence of an increased dose of IFN-γ The results showed that in the presence of IFN-γ , M90T but not BS176 could induce GSDMD cleavage, presumably through the Caspase-11 non-canonical inflammasome (Fig. 1E).
- A more in-depth cell death analysis was done during infection with M90T and BS176 in *Nlrc4*^-/-^ enteroids. It was shown that both strains induce cell death, as both reduce cellular ATP (Fig. 1F). Specifically, apoptosis was shown to occur, as western blots revealed there is an induction of cleaved Caspase-8 and cleaved Caspase-3 following infection (Fig. 1G).
- 2 biological replicates were added to qPCR validation of increased enteroendocrine cell markers following infection with M90T or BS176. It was revealed that M90T and BS176 both significantly increase enteroendocrine cell mRNA (Fig. 6F).
- CXCL1 ELISA has been performed on *Nlrc4*^-/-^ enteroids infected with M90T or BS176. At 4 hours, there was a decrease of CXCL1 in the supernatant of enteroids infected with both strains, confirming our RNA sequencing findings (Fig 5F).
- We performed western blots on WT enteroids treated with LPS and assessed pyroptotic cell death. It was shown that LPS could not enter and trigger GSDMD cleavage even with IFN-γ priming (Fig 2G).
- Colocalization of M90T and BS176 with secretory cells was assessed by immunofluorescence. It was shown that both M90T and BS176 co-localized with goblet cells (Sup. Fig 1A).
- Colocalization of fluorescent beads with secretory cells was assessed by immunofluorescence. It was shown that beads co-localized with goblet cells(Sup. Fig 1B).
- To assess if goblet cells or enteroendocrine cells were the main cell-types responsible for sampling of bacteria, we performed CFUs of M90T or BS176 on *Nlrc4*^-/-^ enteroids pre-treated with drugs to expand the secretory pool of the organoids. We used DAPT, a γ-secretase inhibitor, to expand all secretory cells, and ISX-9 to specifically increase enteroendocrine cells, Tuft cells, and Paneth cells. It was shown that ISX-9 increased CFUs with M90T alone, and not BS176. There was no difference between control enteroids and those treated with DAPT, suggesting secretory cells do not play a major role in sampling bacteria in this setting (Sup Fig 1 C-D).

Point-by-point response to the reviewers’ comments:

# Part I – Summary

## Reviewer #1

**Girardin and colleagues present an interesting study that investigates inflammatory signaling pathways and the impact of S. flexneri on mouse intestinal organoid-derived cells. A strength of this work is the use of a more physiologically relevant model such as mouse intestinal organoids and comparing the results obtained to human epithelial cell lines. However, the authors should take care when making broad statements, as the findings in mouse cells may not be fully recapitulated in human cells. Including a mouse cell line in some of the experiments may be a useful comparison.**

The reviewer raises an interesting point. In figure 1E-F, CFUs of HeLa cells and *Nlrc4*^-/-^ enteroids infected with M90T and BS176 were compared. Specifically, we mentioned:

“ To interrogate whether the bacterial strains exhibited expected infection dynamics, we infected a classic infection model –HeLa cells – with M90T and BS176. As expected, in HeLa cell infections, M90T displayed a significantly higher bacterial load compared to BS176 (Fig. 1E). This is in contrast to the enteroid infection system, where *Nlrc4^-/-^* enteroids internalized both M90T and BS176 at comparable levels (Fig. 1F, 1G), demonstrating differences in bacterial uptake and replication between transformed cells and primary enteroids.”

Considering the reviewer’s point, we realize that there are a few degrees of separation between primary murine enteroids and HeLa cells including their species, the 3D vs 2D conformation of the cells, and the fact that one system is transformed and the other is not. It is incorrect to solely attribute this difference in CFU abundance between M90T and BS176 to the fact that HeLa cells are transformed and enteroids are not. To accommodate this, we propose changing the text to saying:

“We next asked whether, in the absence of NLRC4, M90T would display a marked advantage over BS176 in infecting enteroids, as expected from its virulence plasmid expressing host colonization factors. To address this, we compared bacterial burdens in *Nlrc4*^-/-^ enteroids infected with M90T or BS176 to those observed in a standard cellular infection model, the human HeLa cell line. As expected, M90T achieved higher CFUs than BS176 at every timepoint in HeLa cells (Fig. 2A). In striking contrast, CFU levels were similar between M90T and BS176 in *Nlrc4*^-/-^ enteroids (Fig 2B-C), revealing a major difference in bacterial uptake and replication between this organoid system and classic infection models.” (lines 157-165 in the manuscript)

**The authors have presented some very interesting and novel findings here. The data showing that internalisation and response to an avirulent strain of Shigella is unexpected. As too is the response to bacteria-free supernatants. Another strength of this work is the RNA sequence analysis, which revealed a shift towards more differentiated EEC cells. However, these findings have not been fully validated in qPCR results and require follow up experiments.**

First, we thank the reviewer for their interest in the results mentioned. Regarding qPCR validation, we agree with the comment. We believe this comment is geared towards figure 5F, where the qPCR validation for the increase of enteroendocrine cells did not pass statistical significance with an N of 3. we have added 2 more biological repeats of the experiment, and it now does pass statistical significance.The revised data can be seen in Figure 1E.

**A point to consider is that these experiments have been performed using mouse ileal organoids while the Shigella burden is in the colon. This may impact the findings made, as Shigella may not replicate as efficiently in these cells. The authors should address this as a potential limitation of the work, although the data presented here are very valuable nonetheless, and I believe of interest to a broad audience.**

Yes, that is absolutely correct. Although *S. flexneri* can invade the terminal ileum of the small intestine of humans, The reviewer is correct about the fact that most of the burden still lies within the colon. In lieu of this reviewer comment, we have added text in the discussion section to highlight this limitation:

## “A potential limitation of our study is that the murine small intestinal organoid model may not fully recapitulate human colonic infection, which is the primary site of shigellosis in humans. Indeed, some bacterial effectors may only target human proteins. However, this potential limitation also comes with benefits since our experimental setting allows for better characterizing the underpinnings of host-driven responses in the absence of pathogen-driven manipulation. Nevertheless, future work aiming to validate key findings in human colonic organoids to better reflect the natural tropism of *S. flexneri* will be useful to complement and further validate our findings.” (lines 394-401 in the manuscript)

## Reviewer #2

**In this manuscript, the authors used murine intestinal organoids to study the host response to Shigella flexneri infection. They first confirmed that a Nlrc4-dependent Gsdmd cleavage occurred upon Shigella infection of wt organoids using a virulent strain and suggested that murine organoids uptake bacterial ligands from the virulent strain directly from the supernatant, independently of invasion (Fig. 1). Then, they tried to study the role of Nod1/2 in this infection organoid model (Fig. 2), but concluded that murine organoids are not responsive to Nod1/2 ligands (Fig. 3), making the results of infected Nod1/2 organoid a bit irrelevant. Next, they observed an infection-dependent, but virulence-independent transcriptional change upon infection/treatment of organoids with different Shigella strains/supernatant (Fig. 4). Finally, they suggested that infection led to an enrichment of late enteroendocrine cells in a virulence-independent fashion using RNA-Seq analysis (Fig. 5). The manuscript is well written and many of the data presented in this manuscript are interesting. However, it is quite difficult to connect the dots and most of the points raised by the authors are not supported by enough evidence and remain unanswered at the end of the manuscript. I believe that the manuscript would gain in clarity without the Nod1/2 part and with more data on the uptake/invasion mechanisms and EEC maturation (maybe using a time kinetic). In addition, the addition of a summary figure at the end of the manuscript would help the reader.**

We thank the reviewer for these comments on the structure of the manuscript. Regarding the point of NOD1 and NOD2, see below in the minor issues section for the response. As for the summary figure, we do agree that this would be useful, and we added a summary figure in the final manuscript (Sup. Fig. 5).

# Part II – Major Issues: Key Experiments Required for Acceptance

## Reviewer #1:

**On line 133 the authors state that there are differences in bacterial uptake and replication, however only one timepoint has been shown. A timecourse experiment is required to show replication of S. flexneri in organoids. It is important for interpretation of the model to understand whether the virulent M90T is able to set up a productive infection in these cells. It may be that the CFU represent bacterial uptake rather than replication, and this is why M90T and BS176 have similar values.**

We thank the reviewer for this helpful suggestion. In response, we performed the recommended CFU time-course experiments using S. flexneri M90T and BS176 in murine enteroids and HeLa cells. CFUs were quantified at 1, 2, 4, and 24 hours post-infection (Fig. 2A-B). In HeLa cells, we observed a time-dependent increase in intracellular M90T CFUs, peaking at approximately 4 hours post-infection. As these measurements were obtained using a gentamicin protection assay, the increase in CFUs is consistent with intracellular bacterial replication. In contrast, BS176 CFUs were not recoverable from HeLa cells at any timepoint, indicating that this strain is unable to efficiently enter these cells and therefore does not replicate intracellularly (Fig 2A). In murine *Nlrc4*^-/-^ enteroids, CFU levels of M90T and BS176 were comparable at all timepoints examined. Notably, no substantial increase in bacterial burden was observed over time for either strain, suggesting that bacterial replication is minimal or is balanced by bacterial clearance or death within this cellular system(Fig 2B). At 24 hours post-infection, CFUs for both strains were markedly reduced. We speculate that this decrease is likely due to apoptosis of the enteroids, which results in the release of bacteria into the gentamicin-containing media, as demonstrated later in the manuscript.

**Could it also be possible that bacteria that access the organoid lumen are protected from gentamicin? Testing the uptake of E. coli or labelled beads would also be really useful to understand whether the phenomenon of particle uptake is specific to Shigella.**

We thank the reviewer for this insightful comment. We agree that bacteria accessing the organoid lumen may be protected from gentamicin, as the epithelial cells comprising the enteroids form tight junctions that may limit antibiotic penetration into the luminal space. This possibility has been considered in our interpretation of the CFU data. However, z-stack immunofluorescence images (Fig 2C) demonstrate that at least a subset of bacteria are localized within epithelial cells, as indicated by complete encapsulation within phalloidin-labeled actin, supporting intracellular entry rather than exclusive luminal localization.

To address the reviewer’s suggestion regarding particle specificity, we performed additional experiments using E. coli and fluorescent beads. *Nlrc4*^-/-^ enteroids were infected with E. coli DH5α, and intracellular CFUs were recovered following gentamicin protection assays, indicating bacterial uptake (Fig 2D). Moreover, expression of the afimbrial adhesin AfaE in E. coli did not result in increased CFU recovery, suggesting that uptake is not dependent on CD55-mediated adhesion. Consistent with these findings, immunofluorescence z-stack imaging confirmed intracellular localization of E. coli (Fig 2E)*.*

In addition, transcriptional analysis of *Nlrc4*^-/-^ enteroids infected with E. coli revealed increased expression of the enteroendocrine markers ***Rimbp2*** and ***Myt1*** at 4 hours post-infection, in a manner similar to that observed following infection with M90T and BS176 (Sup. Fig. 4B). These results indicate that the observed uptake and transcriptional response are not specific to S. flexneri. Finally, incubation of enteroids with fluorescent beads also resulted in bead internalization, as assessed by immunofluorescence z-stack imaging, further supporting the capacity of enteroids to internalize particulate material (Fig. 2F).

**What is the lower band in Figure 1H? It could be p20 subunit of GSDMD indicating that there has been cleavage and activation. Other assays for cell death or inflammasome activation are required to understand whether other pathways are activated. For example, caspase 1 cleavage may be occurring here. Furthermore, it is too broad to state on line 145 that ‘other inflammasome pathways…remain inactive in this context’.**

We thank the reviewer for raising this important point and for drawing attention to the lower molecular weight band in Figure 1H. This issue was also raised by another reviewer and had initially been overlooked. In the original experiment, the appearance of a prominent p20 band in the M90T + IFN-γ condition, together with a faint p30 fragment and a concomitant reduction in full-length GSDMD, suggested that inflammasome activation was occurring. To address this more rigorously, we performed additional experiments using four independent biological replicates and optimized detection sensitivity by modifying experimental parameters, including use of a new chemiluminescent detection system. In addition, the IFN-γ concentration was increased from 10 ng/mL to 20 ng/mL to enhance priming. Under these conditions, we observed clear and reproducible cleavage of GSDMD, with accumulation of the p30 fragment specifically in *Nlrc4*^-/-^ enteroids primed with IFN-γ and infected with M90T (Fig. 1E). These data are now included in the revised manuscript. IFN-γ priming was employed to enhance caspase-11–dependent sensing by promoting GBP-mediated LPS accessibility and facilitating caspase-11 engagement. In this context, it is therefore expected that cytosol-invasive bacteria such as M90T, which possesses a functional type III secretion system, can be detected by this pathway following priming.

In addition to inflammasome-associated cell death, we also observe evidence of apoptosis in this infection model independent of IFN-γ priming. Specifically, infection with both M90T and BS176 consistently induces cleavage of caspase-3 and caspase-8 (Fig. 1G). Furthermore, measurements of cellular ATP reveal a reduction following infection with either strain, indicating ongoing cell death despite the absence of canonical NLRC4 inflammasome activation (Figure 1F).

In light of these findings, we have revised the manuscript to avoid overgeneralization and have modified statements made about the non-canonical inflammasome being inactive in this context.

**-The qPCR validation of RNA sequencing presented here is not very robust and does not fully support the statements made by the authors. Other methods to assess inflammatory signalling such as cytokine analysis (Fig 4) or detection of differentiation markers by other methods such as by microscopy or flow cytometry (Fig 5) would further support the claims made by the authors. It would also be useful to know whether there is any cell death in their model, to understand whether the shift to terminally differentiated EECs is a consequence of the bacteria on differentiation or of loss of less differentiated cells**

We thank the reviewer for this constructive comment and agree that additional orthogonal validation was required to strengthen the conclusions drawn from the RNA sequencing data. To address the concern regarding differentiation, we performed immunofluorescence staining and quantitative analysis following infection of *Nlrc4*^-/-^ enteroids with M90T or BS176. We observed an increase in the number of cells expressing late enteroendocrine cell (EEC) markers following infection, particularly with M90T. Specifically, quantification revealed an increase in both chromogranin A (ChgA)–positive cells per organoid and serotonin (5-HT)–positive cells per organoid following M90T infection (Fig 6G-H). In contrast, BS176 did not significantly increase the number of 5-HT–positive cells, although it did increase the number of ChgA-positive cells, a pan-EEC marker (Fig 6G-H). These findings suggest that while both strains can influence EEC differentiation, the invasive strain M90T more robustly promotes differentiation toward enterochromaffin cells.

To provide orthogonal validation of inflammatory signaling observed in the RNA sequencing data (Fig. 5), we analyzed cytokine levels in supernatants from infected *Nlrc4*^-/-^ enteroids. CXCL1 protein levels were quantified by ELISA following infection with M90T or BS176. Consistent with the transcriptional data, CXCL1 levels were reduced in the supernatants of infected enteroids compared to controls, providing proteomic support for the RNA sequencing findings (Fig. 5F).

Finally, we agree with the reviewer that it is important to consider whether the observed shift toward terminally differentiated EECs reflects active changes in differentiation programs or the preferential loss of less differentiated cells due to cell death. As described elsewhere in our responses (lines 243-248), we observe evidence of cell death in this model, including induction of apoptotic markers and reductions in cellular ATP levels following infection with both M90T and BS176, despite the absence of canonical NLRC4 inflammasome activation.

## Reviewer #2:

**The title is misleading as it is not supported by enough evidence in this paper. Only the last figure is related to this point and the data presented derive mostly from one RNA-Seq analysis. They haven’t been confirmed independently. I’d suggest adjusting the title or the content of the manuscript.**

We thank the reviewer for raising this point regarding the title and the strength of the supporting evidence. We agree that the conclusions highlighted in the title must be adequately supported by the data presented. In the revised manuscript, we have added new experimental data directly addressing this point (Fig 6 G-H, Sup Fig. 4B), which provide additional, independent support for the central conclusion reflected in the title. These data complement the RNA-Seq analysis by demonstrating the increase of enteroendocrine cells following infection with different gram-negative bacteria/ bacterial fragments.

**L.136-140: if Caspase 11 is activated and therefore cleaved, shouldn’t the level of full-length caspase 11 decrease? I suggest showing the picture of the full membrane for all caspase 11 western blots to allow the visualisation of cleaved caspase-11 in addition to full-length caspase-11. And in Fig. 1H, the disappearance of uncleaved Gsdmd correlates with the appearance of a 20KDa band in interferon-treated infected organoids. How do the authors explain that? Another Gsdmd antibody could be used to confirm the findings. Additionally, a faint band is seen at for the N-term cleaved Gsdmd at around 35KDa for the Ifng-treated M90T infected organoids.**

We thank the reviewer for raising these important points. This issue was also noted by another reviewer, and we have addressed it in our previous response (see our reply to the related comment). Please refer to lines 226-242 to see our response to this comment. Regarding caspase-11, we agree that activation would be expected to reduce full-length protein levels and/or generate a cleaved fragment. In our experiments, the reduction in full-length caspase-11 is consistently observed across infection conditions; however, we have not been able to resolve a cleaved caspase-11 fragment using the antibody employed here. It is possible that the cleavage product is below the detection threshold or that the antibody is not sufficiently sensitive to this fragment. Of note, it is also possible that caspase-11 activation does not lead to protein cleavage as is observed with other caspases like caspase-1, -3 or -8. Indeed, in a recent study from our lab, we showed that CASP4 (the human homolog of murine caspase-11) cleavage was associated with down-regulation of the protein’s activity rather than serving as a proxy for activation (Bruce et al., *Communications Biology*, 2024) To improve transparency, we have included full-length caspase-11 blots showing the lower molecular weight regions in the revised manuscript.

**Fig. 5: please confirm your findings using immunofluorescence (+ quantification) with known EEC and, if available, late EEC markers.**

We thank the reviewer for this suggestion. We agree that immunofluorescence validation with quantification using known EEC markers is important. In response, we performed immunofluorescence staining and quantification of *Nlrc4*^-/-^ enteroids following infection with M90T or BS176. These experiments included both general (ChgA) and late (5-HT) enteroendocrine cell markers. We observed an increase in ChgA- and 5-HT–positive cells following M90T infection, while BS176 primarily increased ChgA-positive cells. These results confirm the RNA sequencing findings and have been incorporated into the revised Figure 5 and the Results section (Figure 5G-H). **L.142: Shigella with a cytosolic reporter plasmid could be used to see if the bacteria reach the cytosol upon infection. In addition, as the authors suggested cytosolic uptake of PAMPs by the enteroids (L.301, L.305-306), one could assume that LPS uptake could as well occur. A western blot analysis of LPS stimulated organoids, as it has been done with NeedleTox, would be nice.**

These comments were very insightful. This experiment the reviewer proposed revealed that there is some specificity in what the epithelium samples. To address this point we assessed GSDMD cleavage in response to LPS. Purified LPS did not induce GSDMD cleavage at any dose tested, even following IFN-γ priming (Fig 2G). These data suggest that cytosolic sampling in this system may be selective for certain types of bacterial molecules and may exclude highly hydrophobic cargo such as LPS.

**L.307-314: the authors suggested a role for GAPs and EECs in the uptake of PAMPs and bacteria. Using Goblet (e.g. Muc2) and EEC (e.g. Chromogranin A) markers and a time kinetic, it should be possible to observe early bacterial invasion/PAMPs uptake in this organoid model by immunofluorescence. Such mechanistic datasets would strengthen the manuscript.**

We thank the reviewer for this suggestion. To explore the cellular route of bacterial and PAMP uptake, we performed additional immunofluorescence analyses. Initially, we observed that intracellular S. flexneri (both M90T and BS176) and fluorescent beads frequently co-localized with goblet cells, as marked by UEA1 staining (Sup. Fig. 1A-B). This observation raised the possibility that goblet cells may contribute to sampling of bacteria or bacterial products. To test this hypothesis functionally, we expanded the secretory cell compartment using DAPT treatment. However, increasing the goblet cell pool did not alter intracellular CFU recovery for either M90T or BS176, suggesting that goblet cells are unlikely to be the primary route of bacterial entry in this model (Sup. Fig. 1C-D).

We also investigated the potential role of enteroendocrine cells (EECs) by selectively expanding this cell population using a small molecule inducer ISX-9. Expansion of EECs resulted in a significant increase in intracellular CFUs following infection with M90T, but not BS176 (Sup. Fig. 1C-D). While this result is intriguing, it does not definitively demonstrate that EECs mediate bacterial sampling, as the expansion could influence infection through other mechanisms (e.g., altered epithelial physiology or barrier properties). Notably, the lack of a similar effect with BS176 argues against a generalized sampling mechanism and suggests that the phenotype may be specific to the invasive strain.

Taken together, these findings suggest that multiple epithelial cell types may be capable of internalizing particles or bacteria, but the precise cellular route and mechanism of sampling remain to be determined. We have therefore included these observations in the revised manuscript as correlative evidence and have highlighted the need for future mechanistic studies to establish causality.

Part III – Minor Issues: Editorial and Data Presentation Modifications

## Reviewer #1:

**There is no indication of number of biological replicates that have been performed, which may cast some doubt over how reproducible the findings are. This information would be useful throughout the manuscript**

We thank the reviewer for this comment and agree that explicitly stating this throughout the manuscript would provide clarity. We have adjusted each figure caption in the updated manuscript.

**- Error on line 123 ‘ligand cytosolic uptake of the ligand’**

We have removed the extra word.

- **For Fig 2A it is unclear what is meant by ‘relative mRNA expression’ when referring to RNA sequencing results, could the authors please clarify this**

We agree with the reviewer. We were referring to FPKM, so we have changed the axis title to reflect that. The change can be found in figure 3A.

**- Given differences between inflammasome activation and susceptibility to Shigella between humans and mice, the authors should be careful when making broad statements. Eg line 143, ‘mouse’ should be added before ‘intestinal epithelium’ for clarity.**

We have changed the text to reflect this (line 148 in the manuscript).

**- While the rationale for using RIPK2 KO organoids to study NOD signalling is sound, it is an overstatement to say that ‘NOD1 and NOD2 do not alter the transcriptome…’ The authors should modify the language used to reflect that the investigation was of RIPK2-dependent effects.**

We agree with the reviewer that our wording was incorrect. We have changed the wording in the manuscript to state the results are RIPK2-dependent to be more precise.

**- Fig 4A,B and 5C the text is too small**

We have increased the text size on these figures to make them more accessible.

**- Statement on line 231 ‘This finding challenges the prevailing model…’ is an overstatement, as it is well established that inflammatory signalling can be inhibited by pathogens during enteric infection**

We have changed the text to now read:

“Unbiased pathway enrichment analysis further supported this observation: “Hallmark TNFα signaling via NF-κB" was significantly downregulated in M90T-infected enteroids compared to uninfected controls (Fig. 5C, D). This finding highlights an unexpected attenuation of NF-κB signalling below the levels observed in control enteroids during *S. flexneri* infection.” (lines 294-298 in the manuscript)

## Reviewer #2

**Figure 2-3: the authors generated Nlrc4-/-Ripk2-/- mice to generate organoids. However, they concluded that wt organoids do not respond to Nod1/2 ligands in Fig. 3, making the results presented in Fig. 2 a bit irrelevant. These 2 datasets have probably been generated in this order, but as such do not bring much to the main story. I suggest removing them or presenting them at best as supplementary data to keep the flow of the manuscript.**

We thank the reviewer for this suggestion. We agree that the Nod1/2 ligand responsiveness experiments and the generation of Nlrc4^⁻/⁻^Ripk2^⁻/⁻^ organoids are negative findings in the context of the current infection model. However, we believe these data are essential to the manuscript since **NOD1/2 are historically considered key epithelial cytosolic sensors of** Shigella. Demonstrating that NOD1/2–RIPK2 signaling does not contribute to the responses observed in our enteroid system is therefore an important negative result that directly addresses a long-standing assumption in the field. Although the lack of Nod1/2 responsiveness in wild-type organoids may appear tangential, it is critical for interpreting the infection data and for defining the limitations of the enteroid model. Without this information, readers could incorrectly assume that NOD1/2 are functional in this system and therefore may misinterpret the infection phenotypes. For these reasons, we believe that Figures 3–4 provide valuable mechanistic and interpretive context and therefore should remain in the main manuscript. However, we are happy to move some panels to the Supplementary Information if the editors feel this would improve the overall flow, while keeping the key conclusions in the main text.

**Maybe out of scope for this paper as the authors did not use any mouse infection in this manuscript, but are Nlrc4-/-Ripk2-/- mice more or less susceptible to infection than the single ko mice?**

We thank the reviewer for this interesting suggestion. We did not perform in vivo infection experiments in this study, as our focus is on epithelial responses using the enteroid model. To directly assess the epithelial contribution of NLRC4 and RIPK2 in vivo, a conditional knockout approach (*Ripk2*^fl/fl^*Nlrc4*^fl/fl^*Vil1-*Cre+) would be required. These tools are not currently available in our laboratory, and generating the necessary mouse strains is beyond the scope of the present work.

**Fig. 4B: the labelling “increase with” and “decrease with” should be adapted to the conditions. The figure legend is unclear. It sounds as if untreated organoids were compared to supernatant-treated organoids, but it doesn’t fit with what’s mentioned in the text. Please clarify. In addition, it would be nice to show the volcano plots of DE genes between supernatant-treated and untreated organoids to corroborate the conclusion that the effect is specific to infection.**

For figure 5A, we have changed the titles of the arrows so they read “increased with M90T” and “increased in UI”. For figure 5B, we have changed the titles of the arrows, so they read “increased with M90T” and “increased in M90T supernatant”. We have also fixed the error in the figure caption. In addition, we have provided the volcano plot between supernatant-treated and TSB control enteroids in the supplementals (Sup. Fig. 4C).

**Fig. 4C: Organoid infection led as well to the upregulation of many genes. Please analyse those** **genes as well and show the terms associated with these infection-induced genes. The data currently presented are not enough to state that “this finding challenges the prevailing model of inflammatory IEC signalling during enteric infection”. The authors would need to show a GSEA analysis including all up and down regulated genes to make such claim.
Fig. 5B: is this analysis the counterpart of Fig. 4C? If yes, please present the data using the same layout, number of top pathway analysed and criteria (organised by gene ratio in Fig. 4C).**

We thank the reviewer for this suggestion. The analysis of infection-induced genes is already included in the revised manuscript. Specifically, the Hallmark pathway enrichment analysis presented in Figure 5C includes downregulated gene sets, and Hallmark pathway enrichment analysis presented in Figure 6B includes upregulated gene sets. The findings therefore represent a global analysis of both increased and decreased gene expression and do not rely solely on downregulated genes.

To address the reviewer’s request for a standardized presentation, we have reformatted Figure 6B to match the layout and criteria used in Figure 5C. This ensures direct comparability between the analyses.

**Fig. 4E: the authors handpicked only 6 genes to reach their conclusion. It would be better to analyse the difference between the virulent and non-virulent strain using RNA-Seq.**

We thank the reviewer for this suggestion. The primary purpose of Figure 5E was to validate the RNA-seq findings comparing uninfected control organoids with those infected with M90T. The six genes presented were selected because they were among the most significantly downregulated genes in this comparison, and they represent key pathway components identified by the transcriptomic analysis. To provide additional context regarding strain virulence, we also examined the expression of these genes following infection with the avirulent strain BS176.

We agree that a direct comparison of the full transcriptomic responses between M90T and BS176 would provide a more comprehensive view of virulence-dependent transcriptional changes. We think that this analysis is beyond the scope of the current study, but we have noted this as an important future direction.

**L.123, remove the extra “ligand”**

We have edited the manuscript to reflect this change.
