# Supplementary material for: Host response to bacteria induces a shift towards the enteroendocrine cell lineage in the murine enteroid model
Source: PLoS Pathog. 2026 Mar 23;22(3):e1014069. doi: 10.1371/journal.ppat.1014069 (PMC13052877; doi:10.1371/journal.ppat.1014069)
Supplement: Attachment — Submitted filename: Response to reviewers. March 3 2026.docx [file ppat.1014069.s008.docx]

**Response to reviewers**

March 3^rd^, 2026

We would like to sincerely thank the reviewers and the editorial board for their attention and comments on our manuscript. We have incorporated the changes suggested or have discussed them below.

# Part I – Summary

## Reviewer #1

**I commend the authors on their responses to reviewer comments and the additional experiments performed which add value to the manuscript and clarify some of the observations made. I am overall satisfied with responses to my comments but I do still think there are some minor issues with the manuscript.**

We thank the reviewer for their continued interest and attentiveness to the manuscript. We hope the point-by-point below will give the reviewer clarity on their remaining comments.

## Reviewer #2

**The authors have addressed many of my comments and I’m overall satisfied with the current version of the manuscript. I only have minor points that I picked up while reviewing the manuscript.
L.777: add abbreviations used for the UMAP in the figure legend.
L.787: I believe the authors meant to say that Panel C was created with Biorender.
L.802/803: the panels are E and F, not A and B.
L.807: the relative expression of Nod1 is not shown.**

We thank the reviewer for flagging these errors, all of which have been resolved. Please refer to the updated manuscript with track changes to see the changes made in text easily.

# Part II – Major Issues: Key Experiments Required for Acceptance

There were no comments made by either reviewer in this section.

# Part III – Minor Issues: Editorial and Data Presentation Modifications

## Reviewer #1

**1. The authors stated in their responses that figure legends have been updated to include biological replicate information but I still can't find this information.**

We apologize to the reviewer for this mistake. Upon reinspection of the manuscript, biological replicate statements were only made for a few of the figures in the manuscript. Namely, the statement was missing from figure 1, figure 2, and supporting figure 2. We have added text to these figure captions stating that experiments were repeated in at least 3 biological replicates.  **2. Caspase cleavage at baseline levels seems a bit high (Fig 1g), it would be good if the authors could comment on this**

We thank the reviewer for this observation. We agree that caspase cleavage is detectable in the baseline condition. As shown in two additional biological replicates of M90T and BS176 infections in NLRC4 KO enteroids, cleaved Caspase-3 and Caspase-8 is consistently present under basal conditions. We note that prolonged exposure of Western blot membranes can reveal cleavage bands even in unstimulated samples. In addition, a modest degree of baseline epithelial stress is expected in organoid systems. As epithelial cells are continuously shed into the organoid lumen and retained until passaging, some accumulation of apoptotic material can occur despite careful timing of experiments within the culture cycle. Importantly, infection with S. flexneri results in a clear and reproducible increase in caspase cleavage relative to baseline. This induction of apoptosis is further supported by the cellular ATP viability assays shown in Figure 1F, which demonstrate a significant reduction in viability following infection.

**3. For the comment on figure 2G (lines 177-179), the authors state that there was no GSDMD cleavage, but it seems there is with IFNg priming?**

We thank the reviewer for pointing this out. More specifically, we meant to say there is no p30 cleavage induced by LPS. We have modified the text to be accurate. The text now says:

“LPS failed to induce GSDMD p30 cleavage, even following IFN-γ-mediated priming of Caspase-11 (Fig 2G).” (lines 181-182) **4. Line 277, should this be reference to Fig 4H, I instead?

5. some typographical errors in discussion, line 376 'findings' should be 'finding', line 405 'we explores'**

We have implemented the corrections the reviewer has noted in points 4 and 5.

**6. Sup fig 5 is not referred to in the text**

We have added a reference for Sup. Fig. 5 at the end of the results section of the manuscript.
